# Vancomycin and daptomycin minimum inhibitory concentration distribution and occurrence of heteroresistance among methicillin-resistant *Staphylococcus aureus* blood isolates in Turkey

**DOI:** 10.1186/1471-2334-13-583

**Published:** 2013-12-10

**Authors:** Banu Sancak, Server Yagci, Deniz Gür, Zeynep Gülay, Dilara Ogunc, Güner Söyletir, Ata Nevzat Yalcin, Devrim Öztürk Dündar, Ayşe Willke Topçu, Filiz Aksit, Gaye Usluer, Cüneyt Özakin, Halis Akalin, Mutlu Hayran, Volkan Korten

**Affiliations:** 1 Department of Medical Microbiology, Hacettepe University Faculty of Medicine, Ankara, Turkey; 2 Department of Microbiology and Clinical Microbiology, Keciören Training and Research Hospital, Ankara, Turkey; 3 Department of Medical Microbiology, Dokuz Eylul University Medical School, Izmir, Turkey; 4 Department of Medical Microbiology, Akdeniz University Medical School, Antalya, Turkey; 5 Department of Medical Microbiology, Marmara University Medical School, Istanbul, Turkey; 6 Department of Infectious Diseases and Clinical Microbiology, Akdeniz University Medical School, Antalya, Turkey; 7 Department of Medical Microbiology, Kocaeli University Medical School, Kocaeli, Turkey; 8 Department of Infectious Diseases and Clinical Microbiology, Kocaeli University Medical School, Kocaeli, Turkey; 9 Department of Medical Microbiology, Osman Gazi University Medical School, Eskişehir, Turkey; 10 Department of Infectious Diseases and Clinical Microbiology, Osman Gazi University Medical School, Eskişehir, Turkey; 11 Department of Medical Microbiology, Uludag University Medical School, Bursa, Turkey; 12 Department of Infectious Diseases and Clinical Microbiology, Uludag University Medical School, Bursa, Turkey; 13 Department of Preventive Oncology, Hacettepe University Oncology Institute, Ankara, Turkey; 14 Department of Infectious Diseases and Clinical Microbiology, Marmara University Medical School, Istanbul, Turkey

**Keywords:** Vancomycin, Daptomycin, MIC, Heteroresistance, MRSA, Blood isolates

## Abstract

**Background:**

The aim of this study was to determine the distribution of vancomycin and daptomycin MICs among methicillin-resistant *Staphylococcus aureus* (MRSA) blood isolates, the prevalence of heterogeneous vancomycin-intermediate *S. aureus* (hVISA) and the relationship between hVISA and vancomycin MIC values.

**Methods:**

A total of 175 MRSA blood isolates were collected from seven university hospitals in Turkey. All isolates were tested for susceptibility to vancomycin and daptomycin by reference broth microdilution (BMD) and by standard Etest method. BMD test was performed according to CLSI guidelines and Etest was performed according to the instructions of the manufacturer. All isolates were screened for the presence of the hVISA by using macro Etest (MET) and population analysis profile-area under the curve (PAP-AUC) methods.

**Results:**

The vancomycin MIC_50_, MIC_90_ and MIC ranges were 1, 2, and 0.5-2 μg/ml, respectively, by both of BMD and Etest. The daptomycin MIC_50_, MIC_90_ and MIC ranges were 0.5, 1 and 0.125 -1 μg/ml by BMD and 0.25, 0.5 and 0.06-1 μg/ml by Etest, respectively. The vancomycin MIC for 40.6% (71/175) of the MRSA isolates tested was >1 μg/ml by BMD. No vancomycin and daptomycin resistance was found among MRSA isolates. Percent agreement of Etest MICs with BMD MICs within ±1 doubling dilution was 100% and 73.1% for vancomycin and daptomycin, respectively. The prevalence of hVISA among MRSA blood isolates was 13.7% (24/175) by PAP-AUC method. MET identified only 14 of the hVISA strains (sensitivity, 58.3%), and there were 12 strains identified as hVISA that were not subsequently confirmed by PAP-AUC (specificity, 92.1%).

**Conclusions:**

Agreement between BMD and Etest MICs is high both for vancomycin and daptomycin. Daptomycin was found to be highly active against MRSA isolates including hVISA. A considerable number of isolates are determined as hVISA among blood isolates. As it is impractical to use the reference method (PAP-AUC) for large numbers of isolates, laboratory methods for rapid and accurate identification of hVISA need to be developed.

## Background

Increasing number of studies from different regions of the world report reduced susceptibility to glycopeptides among methicillin-resistant *Staphylococcus aureus* (MRSA) [1,2]. There are several studies suggesting incidence of heterogeneous vancomycin-intermediate *S. aureus* (hVISA) parallels the increase of vancomycin minimum inhibitory concentration (MIC) [3-7]. Although the clinical significance of vancomycin-intermediate *S. aureus* (VISA) is well defined, the clinical relevance of hVISA isolates still remains controversial. Several recent studies suggest a relationship between vancomycin treatment failure or a worse clinical outcome and increasing vancomycin MICs [1,2]. Although understanding the changes in glycopeptide susceptibility of MRSA is essential, the vancomycin susceptibility patterns of MRSA isolates in Turkey is still largely unknown. Only a few studies have reported the prevalence of hVISA among MRSA isolates obtained from Turkish hospitals. Therefore, a retrospective analysis of 175 MRSA blood isolates, collected from seven major medical centers was performed in order to determine vancomycin and daptomycin susceptibility patterns, the correlation between vancomycin and daptomycin MICs obtained by broth microdilution (BMD) and Etest, the prevalence of hVISA and VISA among these isolates and the relationship between hVISA and vancomycin MIC values.

## Methods

### Bacterial isolates and antimicrobial susceptibility testing

A total of 175 MRSA blood isolates were collected from seven tertiary-care teaching university hospitals (approximately 30 MRSA strains per centre) in Turkey, which were isolated between 2009 and 2010. These isolates were collected as part of standard patient care and no ethical approval was required for their use. All isolates were tested for susceptibility to vancomycin and daptomycin by reference BMD and by standard Etest. Daptomycin was supplied by Novartis-Turkey and vancomycin analytical powder was commercially purchased (Sigma Chemical Company, St. Louis, MO). MIC determinations for vancomycin and daptomycin were performed according to the Clinical and Laboratory Standards Institute (CLSI) M07 –A8 [8]. Mueller–Hinton broth adjusted to contain physiological levels of calcium (50 mg/L) was used when testing daptomycin. Etest was performed according to the manufacturer’s guidelines using vancomycin and daptomycin Etest strips (bioMérieux, France). The Etest MIC was rounded up to the next highest concentration of BMD for comparison between the Etest and the CLSI MIC value where needed. Reference strains of VISA (Mu50, ATCC 700699), hVISA (Mu3, ATCC 700698), and methicillin- and vancomycin-susceptible *S.aureus* (ATCC 29213) were included as control organisms for the antimicrobial susceptibility tests and population analysis profile-area under the curve (PAP-AUC) analysis. Vancomycin and daptomycin MIC breakpoints proposed by CLSI for BMD method were applied to and used for Etest in order to determine the categorical agreement between the two methods. For determining the percent agreement between BMD and the standard Etest, discrepancies between MIC values of no more than one dilution (± 1 dilution) were calculated. For assessment of categorical agreement, minor error was defined as susceptible (S) or resistant (R) by one method and intermediate (I) by the other. Major error was defined as R according to Etest and S according to BMD, and very major error was defined as S according to Etest and R according to BMD [9]. MIC_50_ and MIC_90_ values were used as parameters for giving results of BMD and Etest methods. The MIC_50_ and MIC_90_ represent the MIC value at which ≥50% and ≥90% of the isolates in a test population are inhibited, respectively.

### Screening for hVISA

All isolates were screened for the presence of the hVISA and VISA phenotype by macro Etest (MET) using a high inoculum (2 McFarland) and PAP/AUC method [10].

The MET was performed as previously described [11]. Briefly, several colonies were picked and suspended in normal saline to obtain a 2.0 McFarland density standard. One hundred microliters of this suspension was evenly spread onto a 90-mm brain heart infusion (BHI) agar plate and allowed to dry. Vancomycin and teicoplanin Etest strips (bioMérieux, France) were applied to the surface of the BHI agar and the plates were incubated at 35°C for 48 h. The plates were then examined and the results were recorded at 24 and 48 h. Zones were read at complete inhibition carefully observing for visual hazy growth and microcolonies. A strain was considered positive for hVISA by MET if microcolonies were detected at ≥8 μg/ml for both vancomycin and teicoplanin or at ≥12 μg/ml for teicoplanin alone. The results were independently interpreted by two investigators.

PAP/AUC was determined according to the Wootton et. al.’s method [10]. Briefly, colonies grown overnight on Columbia agar containing 5% sheep blood were suspended in saline to a density equivalent to a 0.5 McFarland turbidity standard. Dilutions of 10^-3^ (10^5^ cfu/ml) and 10^-6^ (10^2^ cfu/ml) were prepared in saline and 50 μl of bacterial suspensions were inoculated onto BHI agar plates containing 0, 0.5, 1, 2, 2.5, 4 and 8 μg/ml vancomycin. Plates were air dried and incubated for 48 h at 35°C. After incubation at 35°C for 48 h, bacterial colony counts (log10 numbers of CFU/ml) were plotted against the vancomycin concentration (0 to 8 mg/l). The graph obtained was used to calculate AUC. The AUC was calculated for each isolate and divided by the AUC value of the reference strain Mu3. The isolates were identified as hVISA or VISA if the ratio of the AUC of the test isolate to that of the reference strain was ≥0.9 and >1.3 respectively. PAP was the definitive criterion for defining the hVISA phenotype. The performance of MET for detecting hVISA was compared with the PAP-AUC ratio results.

### Statistical analyses

Statistical Package for Social Sciences (SPSS) software was used to analyze the correlation between vancomycin and daptomycin MIC values with the Spearman correlation test.

## Results

A summary of MIC range, MIC_50,_ MIC_90_ and the cumulative vancomycin and daptomycin MIC distributions for MRSA isolates determined by BMD and Etest methods are presented in Table 1.

**Table 1:** **The MIC range**, **MIC**_**50 **,_**MIC**_**90 **_**and the cumulative vancomycin and daptomycin MIC distributions for MRSA isolates determined by BMD and Etest methods**

**Drug**-**Method**	**Cumulative % of isolates with the following MIC (μg/ml):**											**MIC**_**50**_	**MIC**_**90**_	**MIC range**
	**0.06**	**0.094**	**0.125**	**0.19**	**0.25**	**0.38**	**0.5**	**0.75**	**1**	**1.5**	**2**			
**Vancomycin**-**BMD**^**1 **^**(n)**							1		103		71	1	2	0.5-2
**(%)**							0.6		59.4		100			
**Vancomycin**-**Etest (n)**						2	13	44	86	28	2	1	2	0.5-2
**(%)**						1.1	8.6	33.7	82.9	98.9	100			
**Daptomycin**-**BMD (n)**			2		15		97		61			0.5	1	0.125-1
**(%)**			1.1		9.7		65.1		100					
**Daptomycin**-**Etest (n)**	1	12	17	31	49	45	14	6				0.25	0.5	0.06-1
**(%)**	0.6	7.43	17.1	34.9	62.9	88.6	96.6	100						

^1^BMD: Broth microdilution.

The MICs of vancomycin by microdilution were ≤1 μg/ml for 104 (59.4%) isolates and 2 μg/ml for 71 (40.6%) isolates. The distribution of vancomycin MICs among isolates as determined by Etest was given in Table 1. When rounded to the nearest doubling dilution, the overall frequency of MRSA isolates for which the MIC was ≥2 μg/ml was 17.1% (30/175) by Etest.

The overall essential agreement within ± 1 doubling dilution between standard BMD method and Etest method was 100% for vancomycin and 73.1% for daptomycin. No correlation was observed between vancomycin MICs and daptomycin MICs with BMD (r = 0.127, p = 0.095) and Etest (r = 0.119, p = 0.117).

No VRSA or VISA isolates were detected among the isolates tested. The overall rate of hVISA among MRSA blood isolates was 13.7% (24/175). Using the MET, 26/175 (14.9%) isolates met the criteria for hVISA. The MET identified only 14 of the hVISA strains (sensitivity, 58.3%), and there were 12 strains identified as hVISA unconfirmed by PAP-AUC (specificity, 92.1%) (Table 2).

**Table 2:** **Comparison of macro Etest method** (**MET**) **to population analysis profile**-**area under the curve** (**PAP**-**AUC**) **method for detection of hVISA isolates**

**Macro Etest**	**PAP**-**AUC**				**Total**	
	**+**		**-**			
	**n**	**%**	**n**	**%**	**n**	**%**
**+**	14	8	12	6.9	26	14.9
**-**	10	5.7	139	79.4	149	85.1
**Total**	24	13.7	151	86.3	175	100

The frequency of hVISA was MIC dependent irrespective of the method used. The percentage of MRSA isolates that are hVISA increased as the vancomycin MIC increases. Of the 71 isolates with a vancomycin MIC of 2 μg/ml, 16 (22.5%) had the hVISA phenotype, whereas only 8 (7.8%) of the 103 MRSA isolates with a vancomycin MIC of 1 μg/ml were hVISA strains by BMD method. Similarly by Etest, 33.3% (10/30) of the isolates with vancomycin MIC >1 μg/ml showed heteroresistance which was higher than that of the vancomycin MIC ≤1 μg/ml (9.7% [14/145]) (Table 3).

**Table 3:** Number and percentage of hVISA isolates at each vancomycin MIC level among MRSA blood isolates

**Vanco BMD MIC (μg/ml)**	**Number of MRSA**	**Percentage of hVISA among MRSA n (%)**
0,5	1	0
1	103	8 (7.8%)
2	71	16 (22.5%)
**Vanco Etest MIC (μg/ml)**		
0,38	2	0
0,5	13	0
0,75	44	3 (%6.8)
1	86	11 (%12.8)
1,5	28	8 (%28,6)
2	2	2 (%100)

## Discussion

Within the susceptibility range (≤2 μg/ml), several studies report vancomycin MIC increases among MRSA over time (MIC creep) [4,12-14]. Others could not detect such a shift and attribute this phenomenon to the methods used to analyze the data [3,15,16]. Meanwhile, isolates with heteroresistance (hVISA) are emerging and the clinical importance of such isolates is uncertain. Vancomycin treatment failures were reported in patients infected with susceptible isolates with MIC values of 1.5 to 2 μg/ml [1,2,17]. Besides, the presence of VISA and hVISA has been associated with worse clinical outcomes [18-20].

The frequency of MRSA isolates with vancomycin MICs above a certain value such as >1 μg/ml vary widely among studies and may depend on the method used [1,2,21,22]. Our results show the vancomycin MIC for 40.6% of the MRSA strains tested was >1 μg/ml by BMD and 17.1% by Etest. We cannot indicate a MIC creep since an earlier data from the same centers are not available for comparison. The reason for relatively high vancomycin MICs is not clear and may be clonal as suggested by other studies in different settings [13,16].

PAP-AUC method is considered as the gold standard for identifying hVISA. As this approach is time-consuming, labor-intensive, expensive and unsuitable for routine laboratories; multiple screening and detection methods have been investigated for detection of hVISA [7,10,11,20,23,24]. In 2001, Walsh et al. [24] described the MET which detect isolates showing reduced susceptibility to glycopeptides. This method was found superior to other methods and exhibited good sensitivity and specificity compared to PAP-AUC method and has been used for screening or as the definitive test to determine the prevalence of hVISA [6,20,24,25].

In this study, we could not find any VISA, while the prevalence of hVISA was detected as 13.7% (24/175) among MRSA blood isolates by PAP-AUC analysis. The reported worldwide prevalence rates of hVISA differ from 0% to 74% among countries [5,20,26,27]. Even in the same country, there have been conflicting results concerning the prevalence of hVISA. Some of these disparities in frequency could be due to differences in detection methods used in the studies, the study designs and patient populations tested [7,20,27]. In addition, in many studies, the prevalence of hVISA wasn’t confirmed by the PAP-AUC method. Therefore, it is very difficult to compare the results of these studies with ours.

When the results of MET were evaluated, we demonstrated that MET identified only 14 of the hVISA strains (sensitivity, 58.3%), and 12 strains which were characterized as hVISA were not subsequently confirmed by PAP-AUC (specificity, 92.1%). Although the results of initial studies showed that the MET had exhibited high sensitivity and specificity compared with PAP-AUC method, [6,20,24,25] the accuracy of this method varies significantly between different studies [3,7,11,23,24,28]. The clinical significance of isolates which are found as positive by MET but not by PAP-AUC method is unknown. The cause of this discrepancy could be due to the characteristics of the strains or the criteria applied to define hVISA. Another reason for the different sensitivity and specificity values might be the different inoculum size (50 μl, 100 μl, 200 μl or 250 μl) used in different studies [11,20,24,28-30]. Wootton et al., reported the sensitivity and specificity of MET as 76% and 89% when they use 100 μl inoculum volume and 96 and 97% when the inoculum size was 200 μl, respectively [11,24]. In current study we followed the manufacturer's recommended method and used 100 μl inoculum size and found low sensitivity, similar to results reported by some of the studies [3,11,23].

Previous studies have reported that the proportion of hVISA isolates increased as the vancomycin MIC increased [3-7]. Similar to these studies, the percentage of hVISA was higher in the isolates with vancomycin MIC >1 μg/ml than those with vancomycin MIC ≤1 μg/ml irrespective of the testing methods we used.

## Conclusions

This is the first study in Turkey investigating the prevalence of hVISA isolates throughout the country, which were collected from 7 centers. We have demonstrated that the prevalence of hVISA among MRSA isolates recovered from blood cultures in Turkey is high. The hVISA isolates are more common among MRSA isolates with MICs between 1 and 2 μg/ml. Therefore, it is essential to monitor the effectiveness of vancomycin treatment especially when the vancomycin MIC of the isolate is >1 μg/ml.

Because of the ability of screening methods for detection of the hVISA isolates vary significantly, the frequency and the clinical significance of the hVISA phenotype still remains unclear. To date no standardized technique for identifying hVISA phenotype has been established. Though the PAP-AUC method is still considered to be the reference method, it is not practical to measure PAP-AUC ratios for large numbers of isolates, which limits its use in clinical microbiology laboratories. Therefore, it is necessary to develop improved laboratory screening methods for rapid and accurate identification of hVISA.

Etest based methods are suggested as alternative methods for detection of hVISA phenotype as these methods are suitable for using in clinical microbiology laboratories. For that purpose we used MET as a second method, but in our evaluation, this method showed low sensitivity (58.3%) but good specificity (92.1%) similar to recent studies. The reevaluation of currently recommended MET cutoff criteria might be useful for the detection of hVISA isolates in the future. Finally, consistent with many studies, all MRSA isolates including hVISA were found susceptible to daptomycin both by BMD and Etest method. In recent years there were reports which show an association between increasing vancomycin MICs and daptomycin non-susceptibility [30]. We did not observe higher daptomycin MICs among isolates with higher vancomycin MIC values. Therefore, daptomycin could be considered as an alternative agent for the treatment of hVISA-infected patients. Even if non-susceptibility to daptomycin is very rare among MRSA isolates, further surveillance studies are needed detect resistance to this agent in the future.

## Competing interests

Daptomycin Etests were provided by Novartis, Turkey.

## Authors’ contributions

BS and VK participated in the study design. All of the authors contributed to acquisition of data and analyses. BS, SY and VK contributed to discussion of the results and writing of the manuscript. All authors read and approved the final manuscript.

## Pre-publication history

The pre-publication history for this paper can be accessed here:

http://www.biomedcentral.com/1471-2334/13/583/prepub
